# End-tidal carbon dioxide variation after a 100- and a 500-ml fluid challenge to assess fluid responsiveness

**DOI:** 10.1186/s13613-016-0141-9

**Published:** 2016-04-22

**Authors:** Matthias Jacquet-Lagrèze, Florent Baudin, Jean Stéphane David, Jean-Luc Fellahi, Patrick B. Hu, Marc Lilot, Vincent Piriou

**Affiliations:** Service d’Anesthésie Réanimation, Centre Hospitalier Lyon Sud, Hospices Civils de Lyon, 165 Chemin du Grand Revoyet, 69495 Pierre-Bénite, France; Université Claude-Bernard, Lyon 1. Campus Lyon Santé Est, 8 Avenue Rockefeller, 69008 Lyon, France; Service d’Anesthésie Réanimation, Centre Hospitalier Louis Pradel, Hospices Civils de Lyon, 59 Boulevard Pinel, 69500 Bron, France; Irvine’s Department of Anaesthesiology and Perioperative Care, University of California, 333 City Blvd W #2150, Orange, CA 92868 USA

**Keywords:** Fluid responsiveness, End-tidal carbon dioxide, Diagnosis accuracy

## Abstract

**Background:**

EtCO_2_ variation has been advocated replacing cardiac output measurements to evaluate fluid responsiveness (FR) during sepsis. The ability of EtCO_2_ variation after a fluid challenge to detect FR in the context of general anaesthesia has not been investigated. Forty patients were prospectively studied. They underwent general anaesthesia for major surgeries. CO was measured by transoesophageal Doppler, and EtCO_2_ was recorded as well as other haemodynamic parameters [heart rate (HR), mean arterial pressure (MAP), pulse pressure (PP)] at baseline, after 100-ml fluid load over 1 min, and at the end of the 500-ml fluid load. We measured the variation of EtCO_2_ at 100 (ΔEtCO_2_100) and 500 ml (ΔEtCO_2_500), and ROC curves were generated. A threshold for ΔEtCO_2_ to predict FR was determined with receiver operating curves (ROC) analysis. The primary end point was the ability of EtCO_2_ variation after a 500-ml fluid load to diagnose FR.

**Results:**

Fifteen patients (38 %) were fluid responders. ROC analysis showed that for a threshold of 5.8 % (ΔEtCO_2_500), sensitivity was 0.6 IC 95 % [0.33; 0.86] and specificity was 1.0 IC 95 % [1.0; 1.0]. An absolute increase of more than 2 mmHg of EtCO_2_ is specific to diagnose fluid responsiveness (spe = 96 [88–100] %, sens = 60 [33–88] %, AUC = 0.80 [0.96–0.65]). HR, MAP, and PP variations and ΔEtCO_2_100 did not bring information to predict or diagnose FR. During fluid challenge, the correlation between CI variation and EtCO_2_ variation was *r* = 0.566, *p* < 0.001.

**Conclusions:**

During surgery, when alveolar ventilation and CO_2_ production are constant, ΔEtCO_2_500 is fairly reliable to assess FR. When the variation of EtCO_2_ is >5.8 %, all patients were responders, but no conclusion could be done when this variation was <5.8 %. ΔEtCO_2_100 failed to predict FR.

*Trial registration* CPP Lyon Sud Est III ref: 2013-027 B, Number ID RCB: 2013-A00729-36 delivered by the ANSM).

## Background

Excess or lack of fluid can be harmful in the perioperative context. Strategies based on cardiac output optimisation have shown to improve outcome in different kinds of surgeries [1]. The Frank–Starling law implies that the heart of a patient working during the ascending part of the curve will improve cardiac output with an increase in preload: volume responsiveness (VR). Conversely, the heart of a patient working in the range of the plateau of the curve will not improve CO: volume non-responsiveness [2]. Fluid loading is the way to increase preload [3]. Volume responsiveness markers, such as direct cardiac output monitoring or surrogate markers, have been implemented to adjust the amount of fluid to a patient’s needs. Static measures such as mean arterial pressure (MAP), pulse pressure (PP), heart rate (HR), and central venous pressure (CVP) cannot predict fluid responsiveness accurately. Dynamic parameters have been developed and have shown to be accurate [4–6]. Parameters based on heart and lung interactions are limited by their dependence on relatively large tidal volumes, arrhythmias, or the need for an arterial line [7]. Passive leg raising (PLR) is not convenient during surgery. Mini-fluid challenges have been described as a reliable method to predict VR if cardiac output is monitored [8]. Another approach is to perform a fluid challenge and assess a posteriori its effectiveness. Even though some of published studies show that PP, HR, or MAP variations do not give valuable information about VR after volume expansion; a majority of anaesthesiologists still use these parameters to assess VR. This can be explained by the fact that cardiac output monitoring is thought to be too invasive [9]. The end-tidal carbon dioxide (EtCO_2_) correlates with cardiac output in experimental settings [10]. EtCO_2_ variation has been recently used to assess cardiac output variation during PLR and allows one to predict fluid responsiveness of septic patients in intensive care units [11, 12].

Measuring EtCO_2_ might be a reliable method to assess the effect of a fluid challenge in the operating room when cardiac output monitoring is not available. But this strategy has not been prospectively evaluated during general anaesthesia with a population including non-septic patients. The correlation between EtCO_2_ and CI has been shown to be stronger in a context of circulatory insufficiency than in a normal haemodynamic state [10]. The primary end point was the ability of EtCO_2_ variation after a 500-ml fluid load to diagnose fluid responsiveness. Secondary end points were to test whether changes in EtCO_2_ during volume expansion correlate with changes in cardiac output and the ability of the mini-fluid challenge (100 ml) to predict fluid responsiveness.

## Methods

Our institution review board (Comité de Protection des Personnes Lyon Est III ref: 2013-027 B, ANSM Number ID RCB : 2013-A00729-36) approved the study protocol and waived signed informed consent. Inform consent was obtained and notified in the medical record.

### Participants

The study was conducted over a 2.5-month period in 2013 in a secondary care university hospital in Lyon. Participants were those admitted for emergency surgery with a high risk of haemodynamic disorders.

The decision to administer a fluid bolus for volume expansion was left to the discretion of the attending anaesthesiologist.

Inclusion criteria were patient undergoing general anaesthesia with mechanical ventilation and monitored with an oesophageal Doppler. The attending anaesthesiologist decision to administer a fluid bolus for volume expansion was a prerequisite for the inclusion of the patient. The need of oesophageal Doppler monitoring was defined in our setting as patients undergoing high-risk surgeries or high-risk patients. High-risk surgeries were hip fractures, peritonitis, and abdominal haemostatic surgery. High-risk patients were patients with heart failure or significant arteriopathy. Exclusion criteria were patient refusal age <18 years, pregnancy, fluid overload before anaesthesia, patients undergoing laparoscopic surgery, and patients with no available Doppler signals or contraindications to this monitoring technique. All patients meeting the inclusion criteria were included in the study except if they refused to participate if Doppler signal was lacking, or if principal investigators were not available.

### Experimental design

**Fig. 1:**
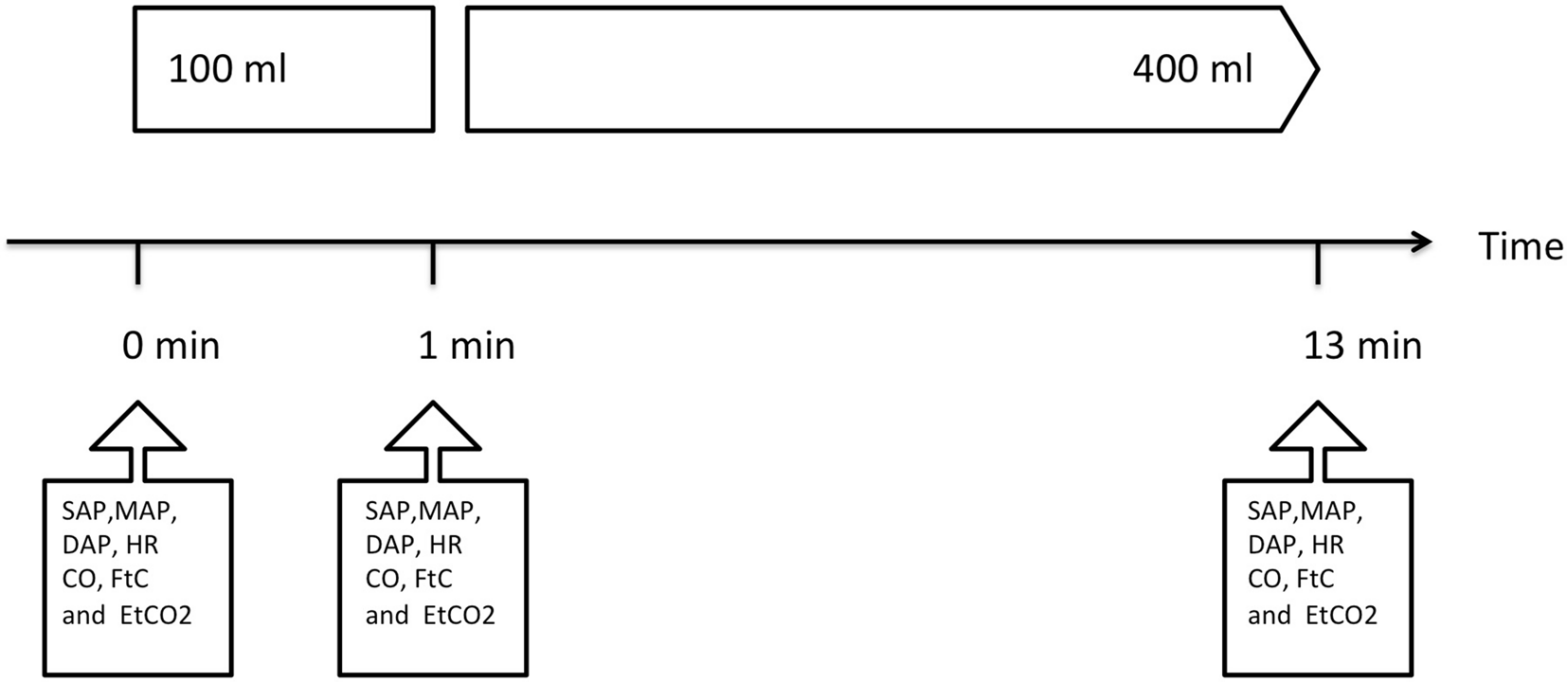
Experimental design. Data were collected at 0, 1, and 13 min. Mean arterial pressure (MAP), systolic arterial pressure (SAP), diastolic arterial pressure (DAP), corrected systolic flow time (Ftc), and end-tidal carbon dioxide (EtCO_2_)

Fluid volume expansion was performed, during the surgery (after the induction of anaesthesia and placement of different monitoring devices). All the data were prospectively recorded, and fluid responsiveness was determined after the fluid bolus and calculated afterthought. The experimental design is detailed in Fig. 1. The manuscript is written to fit with the STARD statement, to allow readers to assess internal and external validity [13].

### Fluid used and administration

Hydroxyethyl starch (*Fresenius Kabi, Germany*) was used. A total of 500 ml divided into one bolus of 100 ml and remaining 400 ml were administered. The first fluid bolus of 100 ml was administered at a rate of 100 ml in 1 min (verified using a stopwatch) using a 50-ml syringe. After 1 min, the remaining 400 ml was administered over 11 min. During the 13 min of fluid administration, there was no modification of the ventilatory settings. Also, no bolus or change in the rate of administration of vasoconstrictor and anaesthetic agents was performed during this period.

### Cardiac output monitoring

Patients were routinely monitored with a transoesophageal Doppler probe (HemoSonic™ 100, ARROW International^®^, 61 mm length, <7-mm section, 5 MHz transducer) which enables recording of continuous descending aortic blood velocity, aortic diameter, aortic ejection volume, aortic blood flow, and other valuable parameters such as acceleration of the flow (Acc) and systolic flow time corrected by heart rate (Ftc). Acc has been described to reflect inotropic change [14], and Ftc has been incorrectly described as a measure of left ventricular preload [15]. An algorithm enables cardiac output computation. The same observer (MJL) performed placement of the probes for all patients. MJL had a good experience with oesophageal monitoring with more than 2-year practice and more than 200 examinations performed. Aortic diameter was visualised and sampled by the transoesophageal probe. The Doppler probe was inserted through the mouth to the place where the maximal aortic blood flow velocity signal could be captured. The quality of the signal was assessed by the visualisation of the two walls of the aorta, and a consistent aortic diameter [16]. Doppler-derived indices were averaged on ten systolic ejections. Cardiac index was calculated as CO divided by body surface. Measurements were recorded before fluid challenges to assessing variability and the least significant change (LSC).

### EtCO_2_ monitoring

The Infinity^®^ EtCO_2_ Microstream SmartPod device was used to monitor EtCO_2_, a side stream device based on infrared absorption of a specific wavelength. This technology provides measures of EtCO_2_. The instantaneous values were recorded at 0, 1, and 13 min.

### Other data and monitoring

Perioperative monitoring included continuous electrocardiogram, pulse oximetry, and non-invasive blood pressure every 5 min. Systolic, diastolic, and mean arterial pressures were recorded. Pulse pressure (PP), defined by systolic minus diastolic pressures, was calculated. Airway pressure, peak pressure, plateau pressure, respiratory rate, and tidal volume were monitored as well as partial inspiratory pressures of O_2_ and CO_2_. The end-tidal anaesthetic agent concentration of desflurane or sevoflurane was monitored to assess deepness of anaesthesia.

### Reference standard

Patients with more than a 15 % increase in cardiac index with a 500-ml fluid load were defined as fluid responders (R), the others being non-responders (NR) This definition is consistent with many diagnosis accuracy studies of fluid responsiveness. The observer was blind from the reference standard as fluid responsiveness was determined after the end of the fluid challenge and was calculated a posteriori.

### Statistical analysis

We calculated with the method of Obuchowsky et al. [17] that 39 patients were needed in order to detect an area under the ROC curve of 0.75 with a power of 0.9 and an alpha risk of 0.05. The ratio between responder and non-responder in the studied population was 0.77. Normal distribution was tested by the d’Agostino–Pearson test. Pairwise comparisons of values were made with the paired Student’s *t* test or Wilcoxon test. The two-tailed Student *t* test or Mann–Whitney *U* test was performed for comparisons between responders and non-responders. In cases of relevancy, data were expressed as variations from baseline computed as the difference between final and baseline value divided by the baseline value and expressed as **Δ**CI and **Δ**EtCO_2_ for CI and EtCO_2_ variation. Correlations were tested by the Spearman method. The relationships between variables underwent linear regression analysis method. Before volume expansion, multiple measurements were recorded during steady haemodynamic and respiratory conditions defined as no need of vasoconstrictor, fluid challenge, and also no respiratory setting modification and no spontaneous breathing detected on the respirator. To assess the reproducibility of the reference standard and the evaluated test, we performed ten successive measures during a stable haemodynamic period. The coefficient of variation was calculated as the standard deviation divided by the mean. The precision was twice the coefficient of variation, and the LSC was computed as 1.96 times the square root time the coefficient of error [18]. The LSC was the minimum change that can be considered as a real change. Data were expressed as mean ± standard deviation (SD) or as median [interquartile range: IQR] when appropriate. ROC curves were built, and AUC was expressed as 95 % confidence interval. Confidence interval was built with the “bootstraps” technique with 2000 repetitions and the same ratio between case and control.

ROC curves were then compared by the Delong test to a 0.5 built ROC curve [19]. Then, ROC curves were used to define three classes of response: negative, inconclusive, and positive. These classes were defined by the author to implement a 10 % diagnosis tolerance in the analysis as it is proposed in a grey zone approach. An EtCO_2_ variation with a value lower than the 90 % sensitivity threshold was defined as negative. An EtCO_2_ variation greater than the 90 % specificity threshold was defined as positive. Remaining EtCO_2_ variations were defined as inconclusive. The proportion of the study population within these limits was calculated. Statistical analysis was performed with R Packages, referenced below [20]. Significant results were defined by a *p* value <0.05.

## Results

**Fig. 2:**
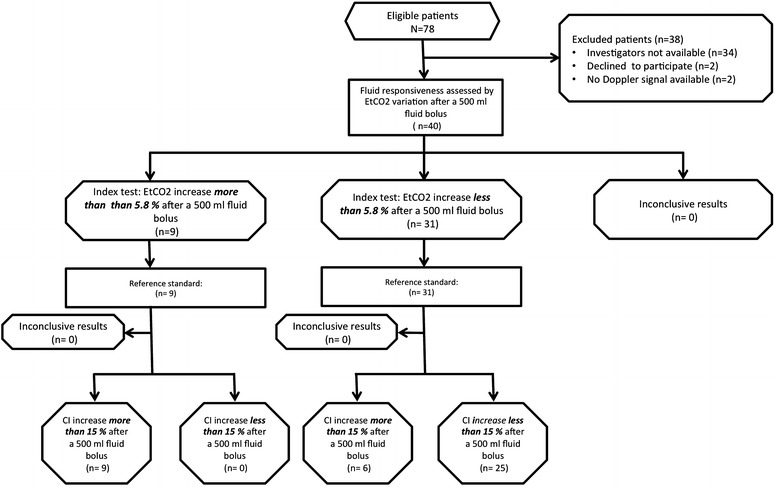
Flowchart of patient recruitment. Cardiac index (CI), end-tidal carbon dioxide (EtCO_2_)

All patients who met inclusion criteria were screened (Fig. 2). Patient characteristics are given in Table 1. The patients were scheduled for orthopaedic (65 %) and abdominal surgery (35 %). All patients underwent general anaesthesia with mechanical ventilation without spontaneous breathing at the time of the study. Twelve patients had surgery in a context of sepsis and nine had a previous history of cardiac failure (Table 1). The precision of EtCO_2_ was 2.2 ± 1.3 %, and the LSC was 3.2 ± 0.2 %. We did not deplore any adverse events as a consequence of the Doppler monitoring or EtCO_2_ measurements. We did not have any missing values of EtCO_2_ or CO at the three different times of the study.

**Table 1:** Characteristics of the population studied

Characteristics	*N* = 40
Age (years)	75 [57–92]
Male sex	17 (43)
Weight (kg)	69 [51–86]
Height (cm)	166 [156–177]
BMI (kg m^−2^)	25 [20–30]
Surgery	
Orthopaedic surgery	26 (65)
General surgery	14 (35)
Sepsis	12 (30)
Antecedent	
ASA score	2.0 [1.6–2.8]
Atrial fibrillation	5 (13)
Cardiac failure	9 (23)
COPD	4 (10)
Treatment prior surgery	
Beta blockers	12 (30)
Other antiarrhythmic agents	5 (13)
Ventilation	
Tidal volume (ml kg^−1^)	6.6 [4.9–8.4]
PEEP (cmH_2_0)	5 [4–7]
FiO_2_ (%)	58 [50–70]
EtCO_2_ (mmHg)	31 [28–34]
SpO_2_ (%)	99 [97–100]
Anaesthesia protocol	
Sufentanil	39 (98)
Propofol TIVA	5 (13)
Halogenated	35 (88)
Curare	16 (40)
Regional anaesthesia	14 (35)
Vasoconstrictors	
Vasoconstritor use	11 (28)
Phenylephrine (µg kg^−1^ min^−1^)	0.2 [0.0–0.3]

Data are expressed as median and [25th–75th] or as number and proportion of patients (*n* = 40). Body mass index (BMI), chronic obstructive pulmonary disease (COPD), positive end-expiratory pressure (PEEP), fraction of inspired oxygen (FiO_2_), end-tidal carbon dioxide (EtCO_2_), arterial oxygen partial pressure (PaO_2_), arterial carbon dioxide partial pressure (PaCO_2_), pulse oxygen saturation (SpO_2_), total intravenous anaesthesia (TIVA), vasoconstrictor (VC)

### Responders and non-responders

**Fig. 3:**
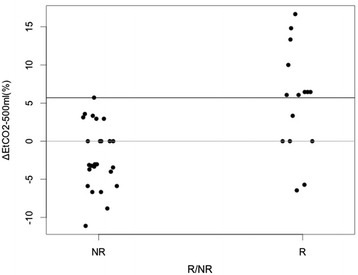
EtCO_2_ variation in responder and non-responder. Variation of end-tidal carbon dioxide after 500 ml (ΔEtCO_2_-500 ml), responders (R) defined as patients who increased cardiac index more than 15 % after fluid expansion and non-responders (NR) defined as patients who increased cardiac index <15 % after fluid expansion

Fifteen patients (38 %) were considered to be fluid responders after a 500-ml bolus. CI increased in all patients by 7.8 [3.1; 20.0] %, in R group by 32 [20; 42] %, and in NR groups, by 3.7 [0; 7.2] %. Distribution of EtCO_2_ variations in responders and non-responders is described in Fig. 3.

### Description of the Variation of CI and EtCO_2_, pulse pressure, heart rate, Ftc, ventilatory change and end-tidal anaesthetic agent concentration during volume expansion

Baseline haemodynamic and respiratory parameters were not significantly different between fluids responders and non-responders (Table 2). No significant differences were found for Vt and minute ventilation between before and after fluid expansion and between both groups at each moment (Table 2). The end of the trial, after the 500-ml volume expansion, showed a significant increase in CI and EtCO_2_ in the responders group and the Ftc increased in both groups (Table 2). The end-tidal anaesthetic agent concentration of desflurane or sevoflurane variation between before and after the fluid challenge was 2.6 [−1.8; 11.4] % in the R group and 0 [0; 11.1] % in the NR group with no significant difference (*p* = 0.9).

**Table 2:** Haemodynamic data before and after a 500-ml fluid load in responders and non-responders

	T0	T500 ml	*p* value
HR (min^−1^)			
R	69 [65; 75]	73 [66; 76]	0.27
NR	69 [61; 77]	70 [63; 74]	0.33
MAP (mmHg)			
R	59 [51; 66]	67 [55; 83]	0.12
NR	63 [58; 67]	64 [56; 72]	0.47
SAP (mmHg)			
R	91 [84; 99]	111 [92; 115]	0.08
NR	95 [88; 107]	95 [90; 112]	0.40
DAP (mmHg)			
R	50 [41; 54]	55 [44; 66]	0.07
NR	50 [47; 56]	53 [45; 60]	0.60
PP (mmHg)			
R	44 [39; 51]	49 [41; 61]	0.16
NR	46 [37; 51]	44 [40; 60]	0.42
Ftc (s/√s)			
R	312 [296; 329]	331 [325; 371]	<0.01
NR	305 [289; 322]	334 [303; 369]	<0.01
EtCO_2_ (mmHg)			
R	31 [30, 32]	33 [31; 33]	0.02
NR	32 [29; 34]	31 [28, 32]^#^	0.04
MV (L/min)			
R	5.1 [4.4; 6.9]	5.2 [4.4; 6.9]	0.52
NR	6.4 [5.8; 7.2]	6.4 [5.7; 7.3]	0.81

Comparison of responders (R) and non-responders (NR): two-tailed Wilcoxon’s rank test was performed between variables to compare before and after fluid expansion. Mann–Whitney *U* test was performed to compare responders to non-responders. Signs ^#^ for significant results comparing R and NR. Significant results comparing before and after fluid expansion are given by *p* value. Heart rate (HR), mean arterial pressure (MAP), diastolic arterial pressure (DAP), systolic arterial pressure (SAP), pulse pressure (PP), corrected systolic flow time (Ftc), cardiac index (CI), end-tidal carbon dioxide (EtCO_2_), and minute ventilation (MV). Data are expressed as median and [25th–75th]

### Correlation between EtCO_2_ and CI

The Spearman correlation test between CI and EtCO_2_ was not significant (*r* = 0.178, *p* = 0.272). However, there was a significant correlation (*r* = 0.566, *p* < 0.001) between CI variation (**Δ**CI) and EtCO_2_ variation (**Δ**EtCO_2_). The slope of the linear regression was 0.172.

### Ability of ΔEtCO_2_ to predict fluid responsiveness after a mini-fluid challenge (100 ml)

The correlation between EtCO_2_ variation and CI variation after a 100-ml fluid challenge was significant: *r* = 0.39, *p* = 0.013. The change in EtCO_2_ after a 100-ml infusion provided an AUC-ROC = 0.74 [0.60; 0.89]; threshold = 3.0 %; sens = 33 [13–60] %, spe = 100 [100–100] %.

### Ability of ΔEtCO_2_ to diagnose fluid responsiveness after a 500-ml volume expansion

**Fig. 4:**
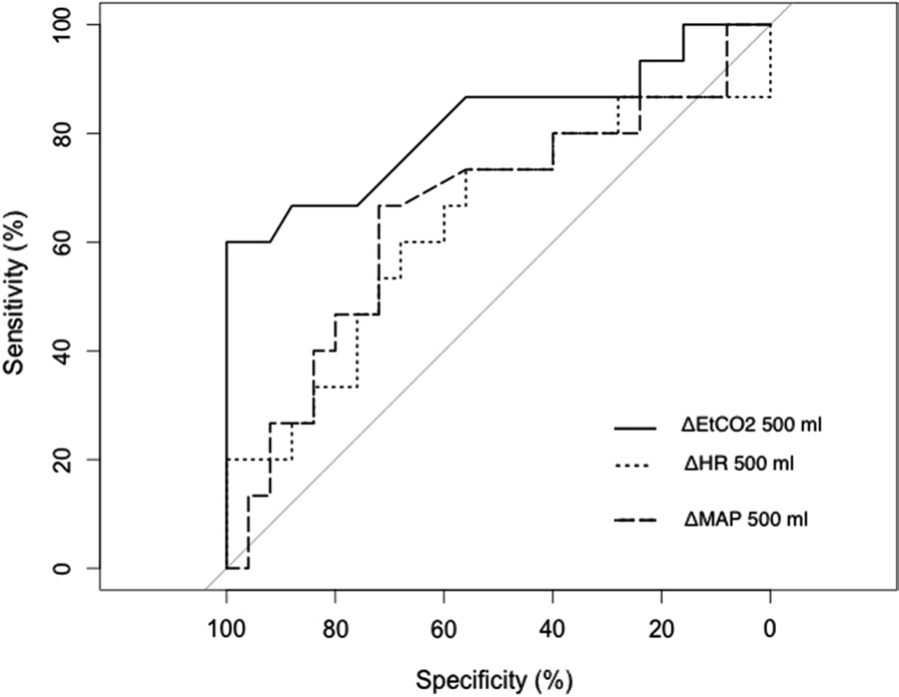
ROC curves of EtCO_2_, MAP, and HR variation to assess fluid responsiveness after a 500-ml fluid load. Mean arterial pressure (MAP), end-tidal carbon dioxide (EtCO_2_), and heart rate (HR) variation after 500-ml fluid load to diagnose responders (R). ΔEtCO_2_, ΔMAP, and ΔHR are the variation % of each hemodynamic parameter after a 500-ml fluid load

The area under the receiver operating characteristic curve (ROC-AUC), sensitivity, specificity, positive predictive value, and negative predictive value likelihood ratios are given in Table 3. **Δ**EtCO_2_ after a 500-ml expansion (**Δ**EtCO_2_500) was able to diagnose fluid responsiveness with a threshold of 5.8 %. In a pragmatic approach, an absolute increase of more than 2 mmHg of EtCO_2_ can diagnose fluid responsiveness (spe = 96 [88–100] %, sens = 60 [33–88] %, AUC = 0.80 [0.96–0.65]). The AUC-ROC for heart rate variation (**Δ**HR_500_), MAP variation (**Δ**MAP_500_), and PP variation (**Δ**PP_500_) was not significantly different from 0.5. ROC curve of **Δ**HR_500_, **Δ**MAP_500_ and **Δ**EtCO_2_500 is shown in Fig. 4.

**Table 3:** Main characteristics of ROC curves built for hemodynamic variables of interest

	AUC	Best thresholds (%)	Compared with AUC = 0.5	Specificity	Sensitivity	PPV	NPV	LR+	LR−	Youden index
varEtCO_2_500	0.82 [0.67; 0.97]	5.89	0.006	1 [1.0; 1.0]	0.6 [0.33 0.87]	1.0	0.81	Infinite	0.4	0.6
varEtCO_2_100	0.74 [0.60; 0.89]	3.0	0.034	1 [1.0; 1.0]	0.33 [0.13; 0.6]	1.0	0.68	Infinite	0.67	0.33
varMAP_500_	0.62 [0.44; 0.83]		0.31							
varPP_500_	0.62 [0.43; 0.82]		0.31							
varHR_500_	0.65 [0.44; 0.82]		0.29							
FtC	0.49 [0.37; 0.67]		0.09							

Mean arterial pressure (MAP), pulse pressure (PP), heart rate (HR), corrected systolic flow time (Ftc), and end-tidal carbon dioxide (EtCO_2_). Data are expressed as variation from baseline (i.e., ΔEtCO_2_ for EtCO_2_ variation) after different volumes of infused fluid: 100 and 500 ml. Area under the curve (AUC), positive predictive value (PPV), negative predictive value (NPV), positive likelihood ratio (LR+), negative likelihood ratio (LR−)

### Clinical ranking of the response with a 10 % diagnosis tolerance

After a 500-ml fluid load, EtCO_2_ variation within the population can be classified as follows: 10 patients had a negative test, 10 patients had a positive test, and 20 patients had an inconclusive test.

## Discussion

This study shows that in a context where alveolar ventilation and CO_2_ production are constant, the change in EtCO_2_ provides information to assess the CI changes during a 500-ml volume expansion. EtCO_2_ variation after a mini-fluid challenge is probably not usable to predict the response to a fluid bolus of 500 ml.

Three factors can explain a change in EtCO_2_: pulmonary blood flow (usually equal to cardiac output), CO_2_ metabolic production, and alveolar ventilation [21]. Naturally, if two of these factors are constant, the change in the third factor can explain the variation of EtCO_2_. Two mechanisms can also explain EtCO_2_ variation. First, a fluid challenge increases venous return and pulmonary blood flow; thus, a greater amount of CO_2_ delivery to the lungs and removal by alveolar ventilation should be observed. Second, a fluid challenge recruits collapsed pulmonary blood capillaries and, therefore, reduces West zones 1 and 2 and increases zone 3 [22]. Consequently, the ventilation/perfusion ratio decreases and dead space is reduced enabling more CO_2_ to be extracted by alveolar ventilation.

Our results are consistent with previously published studies. EtCO_2_ has been described to be able to track CI changes in experimental [10] and clinical settings [23]. Additionally, changes in EtCO_2_ have been used in many clinical situations as cardiac resuscitation for monitoring blood flow generated by precordial compression [24] or assessing the prognosis of cardiac arrest [25, 26]. Two recent studies focusing on patients with septic shock using PLR and volume expansion have shown a significant correlation between changes in EtCO_2_ and CI. Some anaesthesiologists already assess fluid responsiveness by measuring EtCO_2_ variation during a fluid challenge, though this strategy has not been specifically evaluated [27]. As previously described, the correlation between the absolute values of EtCO_2_ and CI was not significant. This is explained by the fact that except for very low CI, EtCO_2_ is mainly influenced by many other factors than CI. Of course, during the short meantime of a volume expansion, these factors can be considered as unchanged.

The threshold value of the EtCO_2_ variation after 500-ml volume infusion was 5.8 %, or an increase of 2 mmHg, which is low, but EtCO_2_ is a very stable variable if no modification of ventilation or cell metabolism occurs, so even a small change can be significant. This idea is reinforced by the fact that the least significant change (LSC) is smaller than the best threshold for a 500-ml load. Moreover, this is about the same threshold as the previously published one [11, 12]. Again, the threshold is near the LSC, and it seems to us that it would be hard to use in the clinical settings for a 100-ml load.

In previous studies, the correlation between changes in EtCO_2_ and CI was stronger during PLR than during volume expansion [11, 12]. Three main explanations can be given.

First, a passive leg raising is shorter than a fluid challenge. This increases the risk of a confounding factor such as ventilation or metabolism variation to be present.

Second, the study protocol was applied during anaesthesia for a surgery and not in a context of circulatory insufficiency. The link between EtCO_2_ and CI is predicted to be weaker when there is no circulatory insufficiency [10].

Third, the preload increase induced by a mini-fluid challenge is inferior compared with a passive leg raising. Our results concerning the mini-fluid challenge are consistent with a study performed recently in a population of septic patients [28]. From our point of view, as long as the fluid challenge is great enough and the infusion time is short, changes in EtCO_2_ could be used as a surrogate for CO changes.

As expected, HR, PP, and MAP variations were unable to predict fluid responsiveness. In a pragmatic approach, with a clinical 10 % tolerance of sensitivity and specificity, as in the grey zone model, we can only conclude for half of the patients. Of course, these results are poorly discriminant. Nevertheless, the discrimination is higher than with parameters mostly used to assess fluid responsiveness as MAP and HR.

We acknowledge that there are some limitations. The variation of EtCO_2_ could be explained by variation in VCO_2_ due to variation in the depth of anaesthesia, but we did not find a significant difference between both groups in the end-tidal anaesthetic agent concentration. We did not use a specific device to monitor the depth of anaesthesia. Nevertheless, end-tidal anaesthetic agent concentration remains the reference to assess depth of anaesthesia [29].

The weak correlation between EtCO_2_ variation, and CI variation after a mini-fluid challenge was largely explained by the imprecision of both measurements: in fact, at that same time the mean variation of CI and EtCO_2_ in the responder group is under the least significant change in the EtCO_2_ and CI.

Our patients benefited from protective mechanical ventilation with positive end-expiratory pressure and low tidal volumes for its positive effect [30]. Consequently, we did not compare our data to pulse pressure variation (PPV) or stroke volume variation (SVV) because we set the tidal volume under 8 ml/kg. These settings make SVV or PPV unable to predict fluid responsiveness [6, 31].

The AUC-ROC after a 500-ml fluid load has a lower confidence interval which is below 0.75. Accordingly, our study has not sufficient power to exclude the fact that the AUC in the population is below this limit. Therefore, we cannot exclude the fact that EtCO_2_ variation after a 500-ml fluid load could be of limited clinical interest according to Ray et al. [32].

To conclude in a setting with a constant alveolar ventilation and CO_2_ production, if no data on cardiac output or pulse pressure variation are available, EtCO_2_ is the only parameter that was discriminant to assess fluid responsiveness. When the variation of EtCO_2_ is >5.8 %, all patients were responders, but no conclusion could be done when this variation was <5.8 %. A 100-ml mini-fluid challenge was not discriminant and cannot be used to predict fluid responsiveness regarding the least significant change in the EtCO_2_. Conversely to widespread belief, MAP, PP, and HR variations were not accurate in predicting fluid responsiveness. The strategy of fluid expansion based on EtCO_2_ variation could be a part of a haemodynamic optimisation protocol regarding its high specificity and its tremendous advantage upon classical cardiac output monitoring to be non-invasive, at no additional cost, and available in all operating rooms.
